# Adaptation and validation of a TB stigma scale for adolescents in Lima, Peru

**DOI:** 10.5588/ijtld.23.0104

**Published:** 2023-10-01

**Authors:** S. S. Chiang, C. Zeng, B. Roman-Sinche, E. Altamirano, C. B. Beckhorn, K. Leon-Ostos, R. Espinoza-Meza, L. Lecca, M. F. Franke

**Affiliations:** 1 Division of Pediatric Infectious Diseases, Department of Pediatrics, Warren Alpert Medical School of Brown University, Providence, RI; 2 Center for International Health Research, Rhode Island Hospital, Providence, RI; 3 Department of Global Health and Social Medicine, Harvard Medical School, Boston, MA, USA; 4 Socios En Salud – Sucursal Perú, Lima, Perú

**Keywords:** depression, adverse childhood experiences, mental health, psychometrics

## Abstract

**BACKGROUND:**

TB-related stigma contributes to poor clinical outcomes and reduced wellbeing for affected individuals. Adolescents may be particularly susceptible to TB-related stigma due to their heightened sensitivity to peer acceptance, yet few studies have evaluated TB-related stigma in this group. Without a validated scale, it remains challenging to measure TB-related stigma in adolescents.

**METHODS:**

We adapted and validated the Van Rie TB Stigma Scale (VTSS) for adolescents on treatment for rifampicin-susceptible TB in Lima, Peru. The modified stigma scale was administered within a larger survey, which measured other psychosocial factors, including depression, adverse childhood experiences (ACEs), and social support. Data analysis included factor analysis, internal consistency, and convergent validity.

**RESULTS:**

From October 2020 to September 2021, 249 adolescents (individuals aged 10–19 years) completed the survey. Preliminary confirmatory factor analysis led to removal of two items. The final 10-item scale demonstrated good internal consistency (Cronbach’s α = 0.82) and adequate model fit (χ^2^/df = 2.0; root mean square error of approximation: 0.06; comparative fit index: 0.94; Tucker-Lewis Index: 0.92: standardized root mean square residual: 0.05). Stigma was positively correlated with ACEs (γ = 0.13), depression (γ = 0.39), and suicidal ideation (γ = 0.27), and negatively correlated with social support (γ = –0.19).

**CONCLUSION:**

This adolescent TB stigma scale may serve as a practical tool to measure TB-related stigma and evaluate the impact of stigma-reduction interventions in adolescents.

## Abstract (fr)

**CONTEXTE:**

La stigmatisation liée à la TB contribue à de mauvais résultats cliniques et à une diminution du bien-être des personnes touchées. Les adolescents sont particulièrement vulnérables à la stigmatisation liée à la TB en raison de leur forte sensibilité à l’approbation de leurs pairs, mais peu d'études ont évalué la stigmatisation liée à la TB dans ce groupe. En l’absence d’une échelle validée, il reste difficile de mesurer la stigmatisation liée à la TB chez les adolescents.

**MÉTHODES:**

Nous avons adapté et validé l'échelle de stigmatisation de la tuberculose de Van Rie (VTSS) pour les adolescents traités pour une tuberculose sensible à la rifampicine à Lima, au Pérou. L'échelle modifiée a été administrée dans le cadre d’une enquête plus large, qui a mesuré d’autres facteurs psychosociaux, y compris la dépression, les expériences négatives de l’enfance et le soutien social. L’analyse des données a porté sur l’analyse factorielle, la cohérence interne et la validité convergente.

**RÉSULTATS:**

D’octobre 2020 à septembre 2021, 249 adolescents (âgés de 10 à 19 ans) ont répondu à l’enquête. Une analyse factorielle confirmatoire préliminaire a conduit à la suppression de deux items. L'échelle finale de 10 items a démontré une bonne cohérence interne (α de Cronbach = 0,82) et un ajustement adéquat du modèle (χ^2^/df = 2,0 ; erreur quadratique moyenne d’approximation : 0,06 ; indice d’ajustement comparatif : 0,94 ; indice de Tucker-Lewis : 0,92 ; résidu moyen quadratique standardisé : 0,05). La stigmatisation était positivement corrélée avec les ACE (γ = 0,13), la dépression (γ = 0,39) et les idées suicidaires (γ = 0,27), et négativement corrélée avec le soutien social (γ = –0,19).

**CONCLUSION:**

Cette échelle de stigmatisation des adolescents face à la TB peut servir d’outil pratique pour mesurer la stigmatisation liée à la TB et évaluer l’impact des interventions de réduction de la stigmatisation chez les adolescents.

TB-related stigma is pervasive and stems from multiple intertwining factors, including fear of TB transmission and associations of TB with other stigmatizing conditions, such as poverty and HIV infection.^1^,^2^ TB-related stigma often involves blaming people with TB, presuming that they became sick because of drug use and/or other perceived moral weaknesses.^1^,^2^ People with TB suffer from anticipated stigma (expectations of being excluded, discriminated against, and/or rejected), enacted stigma (acts of exclusion, discrimination, and/or rejection), and internalized stigma (absorption of negative messages about oneself).^3^ TB-related stigma has been associated with treatment delay and discontinuation.^1^,^2^,^4^–^8^ The harms of stigma also extend beyond treatment, leading to threatened marriage prospects and depression.^9^–^12^ The WHO’s End TB Strategy, which aims to reduce TB incidence by 80% and TB deaths by 90% by 2030, acknowledges the need for stigma reduction to meet these goals.^13^

Adolescents have a high burden of TB disease (approximately 800,000 incident cases each year) and may be particularly susceptible to TB-related stigma due to their heightened sensitivity to peer acceptance.^14^ However, few studies have evaluated TB-related stigma in this group.^3^,^15^,^16^ The available research, limited to qualitative data, has shown that TB-related stigma harms adolescents’ relationships with family members and peers, and contributes to exclusion from schooling and mental health challenges.^3^ However, without a validated scale to measure TB-related stigma in adolescents, it remains challenging to advance this area of research. To address this need, we adapted and validated the Van Rie TB Stigma Scale (VTSS) for adolescents.^17^

## METHODS

### Study setting

Our study was conducted in Lima, the capital of Peru, a middle-income country with an estimated annual TB incidence of 130/100,000 population.^18^ Approximately 70% of patients on TB treatment in Peru receive treatment at community health centers operated by the Ministry of Health.^19^ TB treatment in Lima is administered via facility-based directly observed therapy (DOT), meaning that patients must take their medications under the supervision of providers at health centers. During the COVID-19 pandemic (when this study took place), providers allowed selected patients to take their medications at home under the supervision of a family member or via video DOT after the first 2 weeks of facility-based DOT.

### Study design and participants

From October 2020 to September 2021, we enrolled 252 adolescents with rifampicin-susceptible TB across 71 health centers. Participants were 10–19 years old at treatment initiation and had TB disease affecting any anatomic site. Patients undergoing TB treatment were screened for eligibility using demographic and clinical information available on TB treatment rosters. We invited all eligible adolescents to participate.

Between the third and fifth weeks of TB treatment, participants self-administered-a questionnaire on a tablet computer using the REDCap (Research Electronic Data Capture; Vanderbilt University, Nashville, TN, USA) platform in a private space at a nearby health facility or at home. The questionnaire (Supplementary Box S1) consisted of items to measure demographic and social variables, as well as validated scales to assess depression, adverse childhood experiences, substance use, and treatment self-efficacy. Study personnel remained nearby to answer questions. For each item, participants were given the option of ‘prefer not to respond.’ The questionnaire took 30–45 minutes to complete, and participants were reimbursed for their time with grocery vouchers worth *nuevo soles* (PEN) 30 (approximately USD8.00). At the end of the survey, participants were offered an appointment with the study psychologist to discuss any of the issues mentioned in the questionnaire. Participants with a positive screen for moderate or severe depression were automatically referred to the psychologist.

### Adaptation of the Van Rie TB Stigma Scale

To measure TB-related stigma, Van Rie and colleagues developed an instrument with two subscales, one of which evaluates community perspectives and the other of which evaluates patient perspectives.^17^ The patient perspective scale asks participants to rate their agreement (0 points for strongly disagree to 3 points for strongly agree) using 12 statements about feelings experienced by people with TB. The statements include “Some people who have TB feel alone” and “Some people who have TB are afraid of going to TB clinics because other people may see them there”. We adapted a Spanish version of the patient perspective subscale.^20^ To increase comprehensibility in adolescents, we modified the framing of the items from third-person to first-person. For example, we changed “Some people who have TB feel alone” to “I feel alone because of my TB.” Accordingly, we changed the response categories to ‘never’ (1 point), ‘rarely’ (2 points), ‘sometimes’ (3 points), ‘often’ (4 points), and ‘always’ (5 points). Additionally, because of the low HIV prevalence in Peru (0.4% among people aged 15–49 years^21^), we replaced one of two HIV-related items with “I feel guilty that I got sick with TB because I did not follow a healthy diet.” In Peru, having TB is commonly perceived to be a moral failing of the patient for not eating well or of the caregiver for not feeding the patient well.^4^,^22^ We refined and piloted the entire survey, including the stigma scale, and assessed the acceptability of its length using cognitive interviews with 45 adolescents who previously received TB treatment (see Supplementary Box S2 for details).

### Depression scale

We measured depression using the Patient Health Questionnaire-9 (PHQ-9), which has been validated in Peru and in adolescents.^23^,^24^ The PHQ-9 asks how often (0 points for ‘never’ to 3 ‘points’ for almost every day) the respondent experienced each of nine symptoms in the past 2 weeks. The total score ranges from 0–27 points, with a higher score indicating more severe depression. The PHQ-9 also assesses suicidal ideation (yes or no).

### Adverse childhood experiences

Adverse childhood experiences (ACEs), or potentially traumatic events that occur in childhood, have been associated with worse health outcomes.^25^ We applied a Spanish-language questionnaire, which has been used in adolescents,^26^ that lists 10 ACEs and asks respondents how many they have experienced.

### Treatment self-efficacy scale

We measured self-efficacy for managing medications using a validated scale from the Patient-Reported Outcomes Measurement Information System (PROMIS), which we translated into Spanish.^27^ Using a five-point scale ranging from “I am not at all confident” (1 point) to “I am very confident” (5 points), participants rate four items. The total score ranges from 4 to 20 points, with a higher score indicating greater self-efficacy.

### Social support scale

Based on our qualitative study,^28^ we developed five questions to measure how frequently participants felt supported by parents/guardians, other family members, and friends (Supplementary Table S1). We calculated a total score ranging from 5 to 25 points, with a higher score indicating greater support. This scale showed good internal consistency (Cronbach’s α = 0.76) in the study population.

### Analyses

We evaluated the construct validity of the modified stigma scale using confirmatory factor analysis (CFA). An initial model was specified by assuming that the stigma scale had a single dimension. We evaluated the model fit using the χ^2^ value, root mean square error of approximation (RMSEA; values <0.08 indicate good fit), comparative fit index (CFI; values >0.90 indicate good fit), Tucker-Lewis Index (TLI; values >0.90 indicate good fit), and standardized root mean square residual (SRMR; values <0.06 indicate good fit). As the χ^2^ value increases when the variables in the model increase, we used the ratio of χ^2^ to its degree of freedom (df) to evaluate model fit; a ratio of ≤3 indicates a good model fit. We revised the scale based on the value of modification indices (MI) and our judgement of the contribution of individual items to the measurement of stigma. CFA was based on complete cases. In a sensitivity analysis, we used full information maximum likelihood estimation to handle missing data.

We conducted analyses on the overall study population and subgroups stratified by age (≤15 vs. >15 years old, to account for differences in psychosocial development) and sex. To evaluate internal consistency, we calculated Cronbach’s α. To evaluate convergent validity, we calculated Spearman correlation coefficients between stigma scores and each of the following scales: ACEs, PHQ-9, treatment self-efficacy, and social support. We calculated the point-biserial correlation between suicidal ideation (a binary variable) and stigma. We hypothesized that stigma would be positively associated with ACEs, depression, and suicidal ideation, but negatively associated with treatment self-efficacy and social support.

### Ethics

This study was approved by the institutional review boards of Peru’s National Institute of Health, Lima, Peru; and Rhode Island Hospital, Providence, RI, USA. Participants aged ≥18 years and parents/legal guardians of minors provided written informed consent. Participants aged <18 years provided written assent.

## RESULTS

### Completeness of modified scale items

Of 252 enrolled participants, two withdrew from the study and one declined the questionnaire, leaving a sample size of 249. Of the remaining participants, 231 (92.8%) answered all items on the modified TB stigma scale. Nine (3.6%) participants declined to answer one item, five (2.0%) declined to answer two, and four (1.6%) declined to answer three or more.

### Participant characteristics

Greater proportions of participants identified as male (*n* = 159, 63.9%) or were >15 years old (*n* = 181, 72.7%), reflecting the sex and age distribution of adolescent TB cases in Peru. All participants living with HIV (*n* = 1, 0.4%) and who had been treated previously for TB (*n* = 3, 1.7%) were male and >15 years old. Of 249 participants, 151 (60.6%) received facility-based DOT for the entire treatment, while the remainder were allowed to take medication at home. Table 1 gives participant characteristics and scale scores.

**Table 1:** Characteristics of adolescents undergoing TB treatment in Lima, Peru, stratified by age

Variables	Overall (*n* = 249) median [IQR]	Age groups	
		≤15 years (*n* = 68) median [IQR]	>15 years (*n* = 181) median [IQR]
Age, years,	17 [15–18]	14 [13–15]	18 [17–19]
Female sex, *n* (%)	90 (36.1)	31 (45.6)	59 (32.6)
Living with HIV, *n* (%)*	1 (0.4)	0 (0)	1 (0.6)
Previously treated for TB disease, *n* (%)	3 (1.2)	0 (0)	3 (1.7)
TB stigma (range: 10–50)^†^	22 [17–26]	22 [17–30]	22 [17–26]
Adverse childhood experiences (range: 0–10)^†^	1 [0–3]	1 [0–2]	1 [0–4]
Depression (range: 0–27)^†^	8 [5–11)	9 [5.5–12.5]	8 [5–11]
Treatment self-efficacy (range: 4–20)^†^	11 [11–12]	11 [10–12]	11 [11–12]
Social support (range: 5–25)^†^	20 [16–23]	21 [17–24]	19 [16–23]
Suicidal ideation, *n* (%)	47 (18.9)	18 (26.5)	29 (16.0)

*No participant identified as a gender that was different from their sex assigned at birth.

^†^Measures with a higher score indicate a higher level of stigma, adverse childhood experiences, depression, or social support.

IQR = interquartile range.

### Scale refinement

In preliminary CFA, the following four items had factor loading <0.40: “I feel guilty because I think I got sick with TB because of smoking, drinking alcohol, or using other drugs”; “I keep my distance from others to avoid passing on my TB to them”; “I feel guilty because I have TB and therefore am a burden for my family”; and “I am careful about whom I tell I have TB.” We removed the first two items because of low factor loading and because we judged that they did not contribute to scale validity. The limitation of the first item was the low prevalence of drug use in the study population: of 249 participants, only 14 (5.6%) endorsed drinking alcohol at least twice a month or using other drugs at least once a month. Additionally, we reasoned that because adolescents are instructed by health providers to maintain distance from other people during the first 2 months of TB treatment, the second item may be more reflective of adherence to clinician instructions than stigma.^28^

### Scale scores

We calculated the total score of the remaining 10 items in the scale, with a higher score indicating a higher level of stigma (Table 2). The possible range of scores was 10–50 points. Participants scored between 10 and 45 points. The median stigma scores were consistent among the overall population (22 points, interquartile range [IQR] 17–26) and younger (22 points, IQR 17–30) and older adolescents (22 points, IQR 17–26). Females had higher median scores than males (22 points, IQR 19–29.5 vs. 20 points, IQR 16–26; Supplementary Table S2). Of 249 participants, 42 (16.9%) had scores at or higher than the midpoint of the scale (30 points).

**Table 2:** Internal consistency of the adolescent TB stigma scale and item responses among adolescents on treatment in Lima, Peru, stratified by age

Items	Never (1 point) *n* (%)	Rarely (2 points) *n* (%)	Sometimes (3 points) *n* (%)	Often (4 points) *n* (%)	Always (5 points) *n* (%)
Overall (*n* = 249)*					
1 Scared to tell my family members that I have TB	132 (53.4)	39 (15.8)	52 (21.1)	15 (6.1)	9 (3.6)
2 Scared to go to the health center to get my pills for fear that others will see me	161 (65.2)	39 (15.8)	26 (10.5)	11 (4.5)	10 (4.1)
3 Scared to tell other people I have TB because they may think I have AIDS	147 (60.0)	37 (15.1)	33 (13.5)	16 (6.5)	12 (4.9)
4 Guilty that I got TB because I didn't eat well	57 (23.3)	35 (14.3)	63 (25.7)	35 (14.3)	55 (22.5)
5 Guilty because I have TB and therefore am a burden for my family	119 (48.0)	45 (18.2)	47 (19.0)	24 (9.7)	13 (5.2)
6 Careful about whom I tell I have TB	38 (15.4)	20 (8.1)	51 (20.7)	58 (23.5)	80 (32.4)
7 Scared that I will lose my friends if I tell them I have TB	126 (53.2)	41 (13.7)	32 (13.5)	26 (11.0)	12 (5.1)
8 Scared to tell people outside my family that I have TB	69 (28.2)	47 (19.2)	66 (26.9)	32 (13.1)	31 (12.7)
9 Alone because I have TB	140 (56.5)	44 (17.7)	36 (14.5)	14 (5.7)	14 (5.7)
10 Hurt because of the way others have reacted when they find out I have TB	140 (57.1)	51 (20.8)	31 (12.7)	9 (3.7)	14 (5.7)
Cronbach’s α^†^	0.82				
≤15 years (*n* = 68)*					
1 Scared to tell my family members that I have TB	34 (50.8)	11 (16.4)	13 (19.4)	5 (7.5)	4 (6.0)
2 Scared to go to the health center to get my pills for fear that others will see me	44 (65.7)	10 (14.9)	8 (11.9)	1 (1.5)	4 (6.0)
3 Scared to tell other people I have TB because they may think I have AIDS	37 (55.2)	10 (14.9)	13 (19.4)	1 (1.5)	6 (9.0)
4 Guilty that I got TB because I didn't eat well	20 (29.4)	10 (14.7)	17 (25.0)	9 (13.2)	12 (17.7)
5 Guilty because I have TB and therefore am a burden for my family	37 (55.2)	13 (19.4)	9 (13.4)	6 (9.0)	2 (3.0)
6 Careful about whom I tell I have TB	12 (17.9)	4 (6.0)	14 (20.9)	15 (22.4)	22 (32.8)
7 Scared that I will lose my friends if I tell them I have TB	34 (50.8)	6 (9.0)	9 (13.4)	10 (14.9)	8 (11.9)
8 Scared to tell people outside my family that I have TB	17 (25.4)	12 (17.9)	20 (29.9)	8 (11.9)	10 (14.9)
9 Alone because I have TB	38 (55.9)	9 (13.2)	15 (20.1)	3 (4.4)	3 (4.4)
10 Hurt because of the way others have reacted when they find out I have TB	34 (50.8)	11 (16.4)	12 (17.9)	3 (4.5)	7 (10.5)
Cronbach’s α^†^	0.81				
>15 years (*n* = 181)*					
1 Scared to tell my family members that I have TB	98 (54.4)	28 (15.6)	39 (21.7)	10 (5.6)	5 (2.8)
2 Scared to go to the health center to get my pills for fear that others will see me	117 (65.0)	29 (16.1)	18 (10.0)	10 (5.6)	6 (3.3)
3 Scared to tell other people I have TB because they may think I have AIDS	110 (61.8)	27 (15.2)	20 (11.2)	15 (8.4)	6 (3.4)
4 Guilty that I got TB because I didn't eat well	37 (20.9)	25 (14.1)	46 (26.0)	26 (14.7)	43 (24.3)
5 Guilty because I have TB and therefore am a burden for my family	82 (45.3)	32 (17.7)	38 (21.0)	18 (9.9)	11 (6.1)
6 Careful about whom I tell I have TB	26 (14.4)	16 (8.9)	37 (20.6)	43 (23.9)	58 (32.2)
7 Scared that I will lose my friends if I tell them I have TB	92 (54.1)	35 (20.6)	23 (13.5)	16 (9.4)	4 (2.4)
8 Scared to tell people outside my family that I have TB	52 (29.2)	35 (19.7)	46 (25.8)	24 (13.5)	21 (11.8)
9 Alone because I have TB	102 (56.7)	35 (19.4)	21 (11.7)	11 (6.1)	11 (6.1)
10 Hurt because of the way others have reacted when they find out I have TB	106 (59.5)	40 (22.5)	19 (10.7)	6 (3.4)	7 (3.9)
Cronbach’s α^†^	0.84				

*Three participants ≤15 years old and 15 participants >15 years old) did not answer ≥1 item in the stigma scale.

^†^Calculated using the number of complete cases (65 participants <15 years old and 166 participants >15 years old).

AIDS = acquired immunodeficiency syndrome.

### Internal consistency

Internal consistency was good in the overall study population and subgroups of older and younger adolescents, with Cronbach’s α of 0.82, 0.84, and 0.81, respectively (Table 2). Similarly, consistency was good in males (0.82) and females (0.80) (Supplementary Table S3).

### Convergent validity

Stigma was positively correlated with ACEs (*γ = *0.13), depression (*γ = *0.39), and suicidal ideation (*γ = *0.27), and negatively correlated with social support (*γ = *–0.19) (Table 3). Correlation coefficients were similar in age-stratified subgroups, except for a weaker correlation with social support in younger adolescents. We did not observe any correlation between stigma and self-efficacy in either the overall population or age-stratified subgroups.

**Table 3:** Correlations between adolescent TB stigma scale scores and other key psychosocial indicators among adolescents undergoing TB treatment in Lima, Peru, stratified by age*

	*γ*	*P *value
Overall (*n* = 231)		
Adverse childhood events	0.13	<0.05
Depression	0.39	<0.001
Suicidal ideation	0.27^†^	<0.001
Self-efficacy	0.01	
Social support	–0.19	<0.01
Age ≤15 years (*n* = 65)		
Adverse childhood events	0.17	
Depression	0.36	<0.01
Suicidal ideation	0.25^†^	<0.05
Self-efficacy	–0.07	
Social support	–0.06	
Age >15 years (*n* = 166)		
Adverse childhood events	0.11	
Depression	0.39	<0.001
Suicidal ideation	0.28^†^	<0.001
Self-efficacy	0.05	
Social support	–0.26	<0.01

*Participants who were missing stigma score were excluded from the analysis.

^†^Point-biserial correlation.

Positive correlations between stigma and depression and suicidal ideation were observed in males and females (Supplementary Table S4). ACEs were positively associated with stigma in females (*γ *= 0.23), but not in males (*γ = *0.07). Social support was negatively associated with stigma in males (*γ *= –0.31), but not in females (*γ *= 0.04). There was no correlation between stigma and self-efficacy in gender-stratified subgroups.

### Construct validity

For the revised 10-item scale, the initial model, which assumed independence across scale items, did not meet criteria for good model fit (χ^2^/df = 3.7, RMSEA 0.10, CFI 0.82, TLI 0.76, SRMR 0.07), suggesting model misspecification. According to the values of MI and the conceptual meaning of the stigma scale, we specified the correlations between items 9 and 5; items 1 and 2; items 10 and 9; and items 8 and 6. Adding these correlations improved model fit (χ^2^/df = 2.0, RMSEA 0.06, CFI 0.94, TLI 0.92, SRMR 0.05). The factor loadings across the 10 items ranged from 0.33 to 0.67 (Table 4). Results from the sensitivity analysis did not change the interpretation (Supplementary Table S5).

**Table 4:** Confirmatory factor analysis of adolescent TB stigma scale among adolescents on TB treatment in Lima, Peru (*n* = 231)*

Items	Crude model^†^	Modified model^‡^
1 Scared to tell my family members that I have TB	0.64	0.62
2 Scared to go to the health center to get my pills for fear that others will see me	0.67	0.66
3 Scared to tell other people I have TB because they may think I have AIDS	0.63	0.65
4 Guilty that I got TB because I didn't eat well	0.40	0.39
5 Guilty because I have TB and therefore am a burden for my family	0.35	0.32
6 Careful about whom I tell I have TB	0.41	0.36
7 Scared that I will lose my friends if I tell them I have TB	0.65	0.66
8 Scared to tell people outside my family that I have TB	0.66	0.64
9 Alone because I have TB	0.53	0.47
10 Hurt because of the way others have reacted when they find out I have TB	0.66	0.65
Model fits (indicators and reference)		
χ^2^/df ≤ 3.0	χ^2^/df = 3.5	χ^2^/df = 1.9
*P* ≥ 0.05	*P* < 0.001	*P* = 0.002
RMSEA ≤ 0.06	RMSEA = 0.10	RMSEA = 0.06
CFI ≥ 0.90	CFI = 0.81	CFI = 0.94
TLI ≥ 0.90	TLI = 0.76	TLI = 0.91
SRMR ≤ 0.08	SRMR = 0.07	SRMR = 0.05

*Participants with missing values on the stigma scale were excluded from the analysis.

^†^Model without any modifications.

^‡^Model fit was improved after specifying the correlations between Item 10 and Item 6; Item 1 and Item 2; Item 11 and Item 10; Item 9 and Item 7.

AIDS = acquired immunodeficiency syndrome; df = degrees of freedom; RMSEA = root mean square error of approximation; CFI = comparative fit index; TLI = Tucker-Lewis index; SRMR = standardized root mean square residual.

## DISCUSSION

Our adolescent TB stigma scale demonstrated good validity and consistency, suggesting that it provides a valid, reliable measurement of TB-related stigma among adolescents in Lima. Cronbach’s α was identical to that of the patient perspectives subscale of the original VTSS.^17^ The scale showed good convergent validity; positive correlations with depression and suicidal ideation, and negative correlation with social support were consistent with findings from other studies.^10^,^12^,^29^,^30^ Associations between stigma and treatment self-efficacy have been found in patients with other diseases,^31^,^32^ but we did not observe a correlation. TB treatment delivery is unique in that the standard of care is DOT, rather than self-administration. This approach to treatment delivery may modify the relationship between stigma and treatment self-efficacy that has been observed in other conditions.

The adolescent TB stigma scale performed well in all age- and gender-stratified subgroups. The slightly lower internal consistency in younger adolescents may be due to reduced experience with standardized scales or reduced comprehension of the scale items. Nonetheless, Cronbach’s α for younger adolescents was still high (0.81), suggesting that this scale can be used in adolescents of all ages. Our finding of higher stigma scores in females is consistent with data from other settings and may reflect worse social consequences of TB for females compared to males.^33^ The gender-based differences that we observed in associations between ACEs, social support, and stigma have not been reported previously, and further research is needed to confirm these relationships and explore underlying mechanisms.

This study has limitations. We did not evaluate the test–retest reliability of the adolescent TB stigma scale. We did not evaluate the tool’s ability to measure different categories of stigma, such as anticipated, enacted, and internalized stigma. It is possible that younger adolescents may have more difficulty understanding the items, but we observed good consistency and convergent validity in this subgroup. Another potential limitation relates to generalizability. Several attributes of our adapted scale were targeted to the epidemiological and cultural context of Peru: specifically, the low HIV prevalence and the pervasive belief that TB disease arises from poor eating habits. Moreover, we removed an item about drug use because of low factor loading and low prevalence of drug use in our study population. Therefore, this scale may perform differently in other settings. Nonetheless, the validation of the original VTSS in diverse countries suggests that the adolescent TB stigma scale also may perform well in diverse settings.^10^,^18^,^21^,^34^,^35^

In conclusion, this modified scale may serve as a practical tool to better measure TB-related stigma in adolescents, evaluate risk factors and consequences, and evaluate the impact of interventions to reduce stigma.
