# Supplementary material for: Adaptation and validation of a TB stigma scale for adolescents in Lima, Peru
Source: Int J Tuberc Lung Dis. 2023 Oct 1;27(10):754–60. doi: 10.5588/ijtld.23.0104 (PMC10519385; doi:10.5588/ijtld.23.0104)
Supplement: Supplementary file 1 [file iutld_ijtld_23.0104_supplementarydata1.pdf]

http://dx.doi.org/10.5588/ijtld.23.0104

SUPPLEMENTARY DATA

Adaptation and validation of a TB stigma scale for adolescents in Lima, Peru

Contents:

Supplementary Box S1. English translation of questionnaire . . . . page 2

Supplementary Box S2. Cognitive interviews . . . . page 14

Supplementary Table S1. Items and scoring of the social support scale . . . . page 14

Supplementary Table S2. Key characteristics of adolescents undergoing tuberculosis treatment in Lima, Peru, stratified by gender . . . . page 14

Supplementary Table S3. Adolescent tuberculosis stigma scale internal consistency and item responses among adolescents undergoing tuberculosis treatment in Lima, Peru, stratified by gender . . . . page 15

Supplementary Table S4. Correlations between adolescent tuberculosis stigma scale and other key psychosocial indicators among adolescents undergoing tuberculosis treatment in Lima, Peru, stratified by gender . . . . page 17

Supplementary Table S5. Confirmatory factor analysis (sensitivity analysis) of adolescent tuberculosis stigma scale among all adolescents undergoing tuberculosis treatment in Lima, Peru (N=249) . . . . page 18

Supplementary Box S1. English translation of questionnaire

We would like to invite you to participate in this survey about tuberculosis treatment for adolescents. If you do not understand a word or would like us to clarify a question, please let us know.

In this survey there are questions that might involve some personal topics. I want to remind you that your participation in this survey is completely voluntary. Additionally, all the information that you share with us on this survey will be monitored in a strictly confidential manner (unless you state that you are going to hurt yourself or others). This means that we will not share your answers with your medical providers or your family. All of the information will remain anonymous and be shared only with members of our study team.

A. Household Information

A1a. Please indicate your gender:
• Male • Female • I was born male but I feel female • I was born female but I feel male

A1b. How old are you? _____ years old

A2. Whom do you live with? (Select ALL that apply)
- Mother
- Father
- Stepmother
- Stepfather
- Brother(s)
- Grandparent(s)
- Aunt(s)
- Uncle(s)
- Others; please specify: ___________________________________
- Prefer not to respond

A2b. Number of brothers/sisters younger than 18 years old (not including yourself): ______________

A2c. Number of brothers/sisters 18 years or older (not including yourself): _____________________

A3. Before you had TB, who was working in your family to support your household? (Select all that apply.)
- Mother
- Father
- Stepmother
- Stepfather
- Brother(s)
- Grandparent(s)
- Aunt(s)
- Uncle(s)
- Others; please specify: _____________
- Prefer not to respond

A4. After you began TB treatment, who in your family was working to support your household? (Select all that apply.)
- Mother
- Father

- Stepmother
- Stepfather
- Brother(s)
- Grandparent(s)
- Aunt(s)
- Uncle(s)
- Others; please specify: _____________
- Prefer not to respond

A5a. Are you enrolled in a school/institution/university? (Select ONE.)
- Yes, but not now because of my illness
- Yes, and I am currently taking classes, either in-person or virtually
- No
- Prefer not to respond

A5b. Before the COVID pandemic, how frequently did you feel motivated to attend in-person classes at school or the university?
• Always • Often • Sometimes • Rarely • Never • Prefer not to respond

A5c. How frequently did you feel motivated to attend in-person virtual classes?
- I cannot attend virtual classes because I do not have internet or a computer/smartphone
- Always
- Often
- Sometimes
- Rarely
- Never
- Prefer not to respond

A5d. What are your average grades? In other words, what grades do you most frequently earn?
- AD
- Between AD and A
- A
- Between A and B
- B
- Between B and C
- C
- Prefer not to respond

A6. Do you know what level of education your father completed? (or your stepfather if you consider him as your father)
- None
- Primary
- Secondary
- Institute or university
- I don't know
- Prefer not to respond

A7. Do you know the highest level of education your mother obtained? (or your stepmother if you consider her as your mother)
- None
- Primary
- Secondary
- Institute or university

- I don't know
- Prefer not to respond

A8. Considering that it is necessary to eat 3 times per day for a healthy diet, how often (days per week) have you had to eat fewer meals per day because there was not enough money in the house to buy food?

- Never (0 days/week)
- Rarely (1-2 days/week)
- Sometimes (3-4 days/week)
- Often (5-6 days/week)
- Always (7 days/ week)
- Prefer not to respond

B. TB Symptoms:

B1. What symptoms did you have *before receiving treatment for TB*? (Select ALL that apply.)

- Cough
- Fever
- Chills (intense cold sensation followed by slight tremors of the body)
- Fatigue
- Loss of appetite
- Sweating at night
- Vomiting
- Weight loss
- Hemoptysis (coughing up blood)
- I did not have symptoms
- Other; please specify: ________________________________
- Prefer not to respond

B2. For how long did you had these symptoms before the doctor told you that you have TB?

- Fewer than 15 days
- Between 15 days - 1 month
- 1-2 months
- 2-3 months
- More than 4 months
- Prefer not to respond

B3. What symptoms do you *currently* have? (Select ALL that apply.)

- Cough
- Fever
- Chills (intense cold sensation followed by slight tremors of the body)
- Fatigue
- Loss of Appetite
- Sweating at night
- Vomiting
- Weight loss
- Hemoptysis (losing blood)
- I do not have symptoms
- Other; please specify: ____________________________
- Prefer not to respond

B4. How many pills do you take for your TB each day?

- Fewer de 4 pills

- 4-6 pills
- 7-9 pills
- 10-11 pills
- More than 11 pills
- I do not remember
- Prefer not to respond

B5. Typically, how do you get to the health center to take your medications? (Select ALL that apply.)
- On foot
- Motor-taxi
- Bus
- Taxi
- Private car
- Other; please specify: ____
- Prefer not to respond

B6. Typically, how long does it take you to arrive to health center?
- Less than 5 minutes
- 5-15 minutes
- 15-30 minutes
- 30 minutes-1 hour
- 1-2 hours
- More than 2 hours
- Prefer not to respond

B7. Typically, who pays for your transportation to arrive to the health center? (Select ALL that apply.)
- Mother
- Father
- Stepmother
- Stepfather
- Sibling(s)
- Me
- Others; please specify: ___________________________

B8. What side effects or discomfort do you have or have you had because of the medications? (Select ALL that apply.)
- Rash
- Vomiting
- Headache
- Nausea
- Abdominal pain
- Fatigue
- Others; please specify: ______________________________
- No discomfort
- Prefer not to respond

B9. How often do you experience discomfort as a result of taking your TB medications?
- Never (0 days/week)
- Rarely (1-2 days/week)
- Sometimes (3-4 days/week)
- Often (5-6 days/week)
- Always (7 days/ week)
- Prefer not to respond

B10. How many days of treatment have you missed in the past week?
- 0 days
- 1-2 days
- 3-4 days
- 5-6 days
- I don't remember
- Prefer not to respond

B11. How many days of treatment have you missed in the past 2 weeks?
- 0 days
- 1-2 days
- 3-4 days
- 5-6 days
- I don't remember
- Prefer not to respond

C. Family Relationships

C1. Think of someone that is the person who would have to make legal decisions for you (for example, sign a document). If you are of age (18 or older), think of someone who did this when you were younger than 18. (Select one.)
- Mother
- Father
- Sibling
- Grandparent
- Aunt/uncle
- Guardian (someone who makes decisions for you but is not a relative)

Respond to the following questions, with the person you chose in the previous question in mind. Every time we say "mother/father/guardian," think of this person, and only this person. In the following questions, please respond by selecting ONE of the following options: always, often, sometimes, rarely, never, or prefer not to respond.

C2. My mother/father/guardian treats me with kindness.

C3. I get along well with my mother/father/guardian.

C4. I get along well with other family members.

C5. I confide in my family about the things that happen in my life.

C6. I have secrets that I do not share with my mother/father/guardian.

C7. My mother/father/guardian supports me emotionally with my TB treatment.

C8. Other family members support me emotionally with my TB treatment.

C9. My friends support me emotionally with my TB treatment.

C10. Generally, my mother/father/guardian and I have emotional support from other family members.

C11. Someone from home accompanies me when I go to the health center to receive my pills.

C12. My other family members (outside of my immediate family, in other words people other than

parents or siblings) know that I have TB.

C13. My mother/father/guardian does not like my friends.

C14. I fight with my mother/father/guardian.

C15. My TB illness cases my parents and family members to argue.

C16. Generally, I am happy with my relationship with my mother/father/guardian.

C17. When I go out with friends, my mother/father/guardian lets me decide when I come home.

C18. My mother/father/guardian lets me decide which friends I go out with.

C19. My mother/father/guardian lets me decide what time I go to bed.

C20. My mother/father/guardian lets me decide how I spend my free time.

C21. My mother/father/guardian lets me decide what foods I can eat.

C22. I listen to what my mother/father/guardian tells me (for example, help with housework or chores).

D. My medical care:

D1. I have to wait a long time at the health center before I receive my TB medications.
• Always • Often • Sometimes • Rarely • Never • Prefer not to respond

D2. How many minutes do you normally have to wait before you are given your medications?

- Less than 5 minutes
- 5-15 minutes
- 15-30 minutes
- 30 minutes - 1 hour
- More than 2 hours
- Prefer not to respond

D3. The health worker at the TB program always treats me with respect.
• Always • Often • Sometimes • Rarely • Never • Prefer not to respond

D4. The providers at the health center have clearly explained to me what is TB and what the treatment is like.
• Totally agree • Agree • Somewhat agree • Neutral • Disagree • Prefer not to respond

D5. The physical space of the TB program at the health center is comfortable for adolescents.
• Totally agree • Agree • Somewhat agree • Neutral • Disagree • Prefer not to respond

D6. The providers at the TB program care about my recovery.
• Totally agree • Agree • Somewhat agree • Neutral • Disagree • Prefer not to respond

D7. The providers at the health center observe me when I take my medications.
• Always • Often • Sometimes • Rarely • Never • Prefer not to respond

D8. I am happy with the hours of operation at the TB program.
• Totally agree • Agree • Somewhat agree • Neutral • Disagree • Prefer not to respond

E. Motivation

In the following questions, please respond with one of the following options: Totally agree, agree, somewhat agree, neutral, disagree, or prefer not to respond.

E1. I want to finish my treatment as soon as possible so as not to infect my family members.

E2. I want to finish my treatment as soon as possible so my family no longer has to worry about me.

E3. I want to finish my treatment as soon as possible so I can continue my studies or work.

E4. I want to finish my treatment as soon as possible so I can return to my normal extracurricular activities (go out with friends, play soccer, dance, go skating, etc.).

E5. I am worried that I will have to repeat a school year or some of my studies because of my illness.

F. Knowledge about TB

F1. TB can be completely cured.
• Totally agree • Agree • Somewhat agree • Neutral • Disagree • Prefer not to respond

F2. If I miss some days of my TB treatment, my TB could come back "stronger."
• Totally agree • Agree • Somewhat agree • Neutral • Disagree • Prefer not to respond

F3. If I completely stop taking my TB treatment, my TB could come back "stronger."
• Totally agree • Agree • Somewhat agree • Neutral • Disagree • Prefer not to respond

F4. I have more questions about TB that I would like to be explained to me.
• Totally agree • Agree • Somewhat agree • Neutral • Disagree • Prefer not to respond

F5. I would prefer information about TB to come from: (Select ALL that apply.)

- My parents
- Talks at school/university
- Healthcenter staff
- Other adolescents and youth who have had TB
- TB "advice line" exclusively for adolescents
- Internet (Facebook, Twitter, YouTube, or other websites)
- Other ways; please specify: ________________
- Prefer not to respond

F6. Among the people you know, who has had TB?

- No one
- Mother or father
- Sibling
- Aunt, uncle, and/or cousin
- Grandparent
- Friend and/or neighbor
- Classmate or work colleague
- Prefer not to respond

In the following questions, please respond with ONE of the following options: always, often, sometimes, rarely, never, or prefer not to respond.

F8. I had heard about TB before I got sick.

F9. I have searched for information about TB on the internet.

G. How do I feel about having TB?

G1. I am afraid to tell my family members that I have TB.
• Always • Often • Sometimes • Rarely • Never • Prefer not to respond

G2. I am afraid to go to the health center to get my pills because I am afraid others will see me.
• Always • Often • Sometimes • Rarely • Never • Prefer not to respond

G3. I am afraid to tell people that I have TB because they might think that I also have AIDS.
• Always • Often • Sometimes • Rarely • Never • Prefer not to respond

G4. I feel guilty because I think I got sick with TB because I did not eat well.
• Always • Often • Sometimes • Rarely • Never • Prefer not to respond

G5. I feel guilty because I think I got sick with TB because of smoking, drinking alcohol, or using other drugs.
• Always • Often • Sometimes • Rarely • Never • Prefer not to respond

G6. I feel guilty about getting TB because I am a burden for my family.
• Always • Often • Sometimes • Rarely • Never • Prefer not to respond

G7. I am careful when choosing whom to speak to about my TB.
• Always • Often • Sometimes • Rarely • Never • Prefer not to respond

G8. I might lose my friends if I tell them I have TB.
• Always • Often • Sometimes • Rarely • Never • Prefer not to respond

G9. I am afraid to tell people who are not family members that I have TB.
• Always • Often • Sometimes • Rarely • Never • Prefer not to respond

G10. I feel alone because of my TB.
• Always • Often • Sometimes • Rarely • Never • Prefer not to respond

G11. I feel hurt by the way people react when they find out that I have TB.
• Always • Often • Sometimes • Rarely • Never • Prefer not to respond

G12. I keep my distance from others to avoid passing on my TB to them.
• Always • Often • Sometimes • Rarely • Never • Prefer not to respond

H. Mood and emotions

The following questions are about your feelings and emotions. How often have you felt each of the following symptoms during the PAST TWO WEEKS? For every symptom, select the option that best describes how you have felt.

H1. Have you ever felt down, depressed, irritable, or hopeless?
• Never • Some days • More than half the days • Almost every day

H2. Have you felt little interest or pleasure in doing things?
• Never • Some days • More than half the days • Almost every day

H3a. Have you had problems falling asleep or staying asleep?
• Never • Some days • More than half the days • Almost every day

H3b. Or by contrast, have you been sleeping too much?
• Never • Some days • More than half the days • Almost every day

H4. Have you ever felt tired or with little energy?
• Never • Some days • More than half the days • Almost every day

H5a. Have you had a lowered appetite or have you lost weight?
• Never • Some days • More than half the days • Almost every day

H5b. Or, by contrast, have you eaten excessively?
• Never • Some days • More than half the days • Almost every day

H6. Have you felt bad about yourself or have you thought of yourself as a failure, or someone who has disappointed yourself or your family?
• Never • Some days • More than half the days • Almost every day

H7. Have you had difficulties concentrating in certain activities, like school work, reading, or watching television?
• Never • Some days • More than half the days • Almost every day

H8a. Have you ever moved or talked so slowly that other people have taken notice?
• Never • Some days • More than half the days • Almost every day

H8b. Or, by contrast, have you been so restless that you haven't been able to stop moving more than usual?
• Never • Some days • More than half the days • Almost every day

H9. Have you ever thought that it would be better to be dead or have you thought of hurting yourself in some way?
• Never • Some days • More than half the days • Almost every day

I. Alcohol consumption

It is important that we ask you certain questions about your consumption of alcohol. Your responses will be confidential, so please be honest.

In the past 12 months:

I1. How often do you drink alcoholic beverages?
- Never
- Once or less per month
- From 2-4 times per month
- From 2-3 times per week
- 4 or more times per week
- Prefer not to respond

I2. How many alcoholic drinks do you normally have on a typical day?
- 1 to 2
- 3 to 4
- 5 to 6
- 7 to 9
- 10 or more
- Prefer not to respond

I3. How often do you have 6 or more alcoholic beverages in only one day?
• Never • Monthly • Weekly • Almost daily • Daily • Prefer not to respond

I4. How often in the past year have you been unable to stop drinking once you started?
• Never • Monthly • Weekly • Almost daily • Daily • Prefer not to respond

I5. How often in the past year have you not been able to do what was expected of you because you drank?
• Never • Monthly • Weekly • Almost daily • Daily • Prefer not to respond

I6. How often in the past year have you had to drink alcohol in order to recover after drinking too much the previous day (or to avoid a hangover)?
• Never • Monthly • Weekly • Almost daily • Daily • Prefer not to respond

I7. How often in the past year have you felt remorse or guilt after drinking?
• Never • Monthly • Weekly • Almost daily • Daily • Prefer not to respond

I8. How often in the past year have you been unable to remember what happened the night before because you had been drinking?
• Never • Monthly • Weekly • Almost daily • Daily • Prefer not to respond

I9. Have you or another person gotten injured because you had been drinking?
- No
- Yes, but not in the past year
- Yes, in the past year
- Prefer not to respond

I10. Has someone you know (friend, doctor, or professional) been concerned about your consumption of alcohol or has suggested that you stop drinking?
- No
- Yes, but not in the past year
- Yes, in the past year
- Prefer not to respond

J. Drug use

J1. At least once have you taken any of these drugs (without medical prescription)? PLEASE MARK ALL THAT YOU HAVE TAKEN AND HOW OFTEN.

One of the following options will be selected for the items below:
- Never
- 1-2 times
- Every month
- Every week
- Daily or almost daily
- Prefer not to respond

a. Tobacco (cigarettes, pipes, cigars, chewing tobacco, pipe, etc.)

b. Cannabis (marijuana, hashish, etc.)

c. Cocaine (coca, crack, etc.)

d. Amphetamines or other stimulants (speed, ecstasy, ice, Ritalin, Dexedrine, slimming pills, methamphetamine, etc.)

e. Inhalants (glues, terocal, gasoline / naphtha, glue, solvents, Poppers etc.)

f. Tranquilizers or sleeping pills (valium / diazepam, Trankimazin / Alprazolam / Xanax, Orfidal / Lorazepam, Rohipnol, Librium, Ativan, GHB, Nembutal, Seconal, Phenobarbital, etc.)

g. Hallucinogens (LSD, acids, ketamine, mezcalin, PCP, angel powder, etc.)

h. Opioids (heroin, methadone, codeine, morphine, dolantine / pethidine, OxyContin, Darvon, Vicodin, Dilaudid, Demerol, Lomotil, Percodan, etc.)

i. Others; please specify: ___________________________

J2. In the past 12 months:

For the questions below, one of the following will be selected:
- No
- Yes
- Prefer not to respond

J2a. Have you felt the need or desire to take the drugs you mentioned that you were unable to resist?

J2b. Have you tried to not take or have you had difficulties with stopping before becoming intoxicated?

J2c. When you take less drugs or do not take them at all, do you feel symptoms from withdrawal (pain, shaking, fever, weak feelings, diarrhea, nausea, sweating, elevated heartbeat, difficulty sleeping, or feeling agitated, irritable, or depressed)? Or, have you taken another substance to avoid feeling these withdrawal symptoms?

J2d. Have you noticed that you have to take a higher dose of drugs to get the same effects has before?

J2e. Have you reduced your activities activities (in your free time, at rest, or daily activities) because you have been taking drugs?

J2f. Have you continued taking the drugs you mentioned even though you know they result in physical and psychological problems?

J3. In the past 12 months:

For the questions below, one of the following will be selected:
- No
- Yes
- Prefer not to respond

J3a. Since you last took the drug(s) you mentioned, have you had health problems like accidental overdose, chronic cough, convulsions, infections, problems with your liver, or an injury?

J3b. Since you started taking drugs, have you had psychological problems, like not being interested in anything, feeling sad, not trusting other people, having thoughts of being persecuted or other strange thoughts?

J3c. Have you had problems at school, at work, or at home because of taking drugs?

Sections K and L were not relevant to the current analysis and have not been included.

Many children and adolescents have stressful experiences that may impact their health and well-being. Read the following statements. Tell us which ones which may apply to you by writing the total number in the corresponding box. Do NOT mark nor indicate the specific statements that apply to you.

M1. Of the following statements, HOW MANY are applicable to you?
Write the total in the box: ________________________________ (0 – 10 points)

At some point in time since you were born:

- Your parents or guardians divorced or separated.
- You lived with someone that was in jail or prison.
- You lived with someone that suffered from depression, had a mental illness, or attempted suicide.
- You saw or heard people you trust hurt themselves or threaten to hurt themselves.
- A person that you trust has been rude to you, insulted you, humiliated you, or criticized you in a way that scared you or made you afraid that you would be harmed in a physical manner.
- Someone touched you in your private parts or asked you to touch their private parts in a sexual manner that you did not want, against your will, or that made you feel uncomfortable.
- You have lacked food, clothing, a place to live or someone to protect you.
- Someone pushed, grabbed, slapped, threw something or hit you with enough force that it left a mark or bruise.
- You lived with someone that had an alcoholic or drug problem.
- You have felt that no one is there to support, love, or protect you.

M2. If you would like to speak with a study psychologist about any of the issues mentioned in this survey, please select “yes” below.

- Yes
- No
- Prefer not to respond

Supplementary Box S2. Cognitive interviews

We refined the survey – including the adapted stigma scale – through one-on-one cognitive interviews with 45 adolescents who had completed treatment for rifampicin-susceptible TB disease in Lima in the preceding 12 months. From January to March 2020, we carried out nine rounds of five cognitive interviews each. In these interviews, participants were read each item, asked to paraphrase the question back to the interviewer, provide and explain their answer, and make suggestions about how to optimize clarity. After each round, the research team refined the questionnaire, with the goal of optimizing comprehension by our target population while providing the desired information. Participants also were asked for their feedback on the length of the questionnaire. Items were eliminated to make the survey length acceptable to adolescents. The final round of interviews served as a pilot. Participants ≥18 years old and parents/guardians of minors provided written informed consent. Participants <18 years old signed assent forms. All participants were reimbursed for their time with grocery gift cards valued at 45 *nuevo soles* (equivalent to approximately USD 13.50).

Supplementary Table S1. Items and scoring of the social support scale

**Items**	**Responses (number of points)**
1. My mother/father/guardian supports me emotionally with my TB treatment	Never (1)
2. Other family members support me emotionally with my TB treatment	Rarely (2)
3. My friends support me emotionally with my TB treatment	Sometimes (3)
4. Generally, my mother/father/guardian and I always have had emotional support from other family members	Often (4)
5. Someone from home accompanies me when I go to the health center for my medications	Always (5)

Abbreviations: TB, tuberculosis.

Supplementary Table S2. Key characteristics of adolescents undergoing tuberculosis treatment in Lima, Peru, stratified by gender

Variables (Median, IQR)*	Overall population N=249 n (%)	Gender	
		Male N=159 n (%)	Female N=90 n (%)
Age	17 (15-18)	17 (16-19)	17 (15-18)
Living with HIV (n, %)	1 (0.4)	1 (0.6)	0 (0)
Previously treated for TB disease (n, %)	3 (1.2)	3 (1.9)	0 (0)
Tuberculosis stigma (range: 10~50)†	22 (17-26)	20 (16-26)	22 (19-29.5)
Adverse childhood experiences (range: 0~10)†	1 (0-3)	1 (0-3)	1 (1-4)
Depression (range: 0~27)†	8 (5-11)	7 (4-11)	9 (6-13)
Self-efficacy (range: 4~20)†	11 (11-12)	11 (11-12)	11 (11-12)
Social support (range: 5~25)†	20 (16-23)	20 (16-23)	20 (16-23)
Suicidal ideation (n, %)	47 (18.9)	24 (15.1)	23 (25.6)

Abbreviations: HIV, human immunodeficiency virus; IQR, interquartile range; TB, tuberculosis.
*Unless otherwise specified
† A higher score indicates a higher level of stigma, adverse childhood experiences, depression, or social support.

Supplementary Table S3. Adolescent tuberculosis stigma scale internal consistency and item responses among adolescents undergoing tuberculosis treatment in Lima, Peru, stratified by gender

Items	Never (1 point)	Rarely (2 points)	Sometimes (3 points)	Often (4 points)	Always (5 points)
**Overall (N=249)**†					
1. Scared to tell my family members that I have TB	132 (53.4)	39 (15.8)	52 (21.1)	15 (6.1)	9 (3.6)
2. Scared to go to the health center to get my pills for fear that others will see me	161 (65.2)	39 (15.8)	26 (10.5)	11 (4.5)	10 (4.1)
3. Scared to tell other people I have TB because they may think I have AIDS	147 (60.0)	37 (15.1)	33 (13.5)	16 (6.5)	12 (4.9)
4. Guilty that I got TB because I didn't eat well	57 (23.3)	35 (14.3)	63 (25.7)	35 (14.3)	55 (22.5)
5. Guilty because I have TB and therefore am a burden for my family	119 (48.0)	45 (18.2)	47 (19.0)	24 (9.7)	13 (5.2)
6. Careful about whom I tell I have TB	38 (15.4)	20 (8.1)	51 (20.7)	58 (23.5)	80 (32.4)
7. Scared that I will lose my friends if I tell them I have TB	126 (53.2)	41 (13.7)	32 (13.5)	26 (11.0)	12 (5.1)
8. Scared to tell people outside my family that I have TB	69 (28.2)	47 (19.2)	66 (26.9)	32 (13.1)	31 (12.7)
9. Alone because I have TB	140 (56.5)	44 (17.7)	36 (14.5)	14 (5.7)	14 (5.7)
10. Hurt because of the way others have reacted when they find out I have TB	140 (57.1)	51 (20.8)	31 (12.7)	9 (3.7)	14 (5.7)
Cronbach's alpha‖			*0.82*		
**Males (N=159)**†					
1. Scared to tell my family members that I have TB	97 (61.4)	21 (13.3)	28 (17.7)	7 (4.4)	5 (3.2)
2. Scared to go to the health center to get my pills for fear that others will see me	113 (71.5)	15 (9.5)	20 (12.7)	4 (2.5)	6 (3.8)
3. Scared to tell other people I have TB because they may think I have AIDS	104 (67.1)	19 (12.3)	18 (11.6)	8 (5.2)	6 (3.9)
4. Guilty that I got TB because I didn't eat well	40 (25.5)	20 (12.7)	43 (27.4)	21 (13.4)	33 (21.0)
5. Guilty because I have TB and therefore am a burden for my family	76 (47.8)	29 (18.2)	28 (17.6)	19 (12.0)	7 (4.4)

6. Careful about whom I tell I have TB	32 (20.3)	13 (8.2)	34 (21.5)	34 (21.5)	45 (28.5)
7. Scared that I will lose my friends if I tell them I have TB	87 (57.2)	23 (15.1)	20 (13.2)	15 (9.9)	7 (4.6)
8. Scared to tell people outside my family that I have TB	53 (34.0)	32 (20.5)	42 (26.9)	15 (9.6)	14 (9.0)
9. Alone because I have TB	104 (65.4)	19 (12.0)	19 (12.0)	8 (5.0)	9 (5.7)
10. Hurt because of the way others have reacted when they find out I have TB	97 (61.4)	33 (20.9)	18 (11.4)	5 (3.2)	5 (3.2)
Cronbach's alpha‖			*0.82*		
**Females (N=90)†**					
1. Scared to tell my family members that I have TB	35 (39.3)	18 (20.2)	24 (27.0)	8 (9.0)	4 (4.5)
2. Scared to go to the health center to get my pills for fear that others will see me	48 (53.9)	24 (27.0)	6 (6.7)	7 (7.9)	4 (4.5)
3. Scared to tell other people I have TB because they may think I have AIDS	43 (47.8)	18 (20.0)	15 (16.7)	8 (8.9)	6 (6.7)
4. Guilty that I got TB because I didn't eat well	17 (19.3)	15 (17.1)	20 (22.7)	14 (15.9)	22 (25.0)
5. Guilty because I have TB and therefore am a burden for my family	43 (48.3)	16 (18.0)	19 (21.4)	5 (5.6)	6 (6.7)
6. Careful about whom I tell I have TB	6 (6.7)	7 (7.9)	17 (19.1)	24 (27.0)	35 (39.3)
7. Scared that I will lose my friends if I tell them I have TB	39 (45.9)	18 (21.2)	12 (14.1)	11 (12.9)	5 (5.9)
8. Scared to tell people outside my family that I have TB	16 (18.0)	15 (16.9)	24 (27.0)	17 (19.1)	17 (19.1)
9. Alone because I have TB	36 (40.5)	25 (28.1)	17 (19.1)	6 (6.7)	5 (5.6)
10. Hurt because of the way others have reacted when they find out I have TB	43 (49.4)	18 (20.7)	13 (14.9)	4 (4.6)	9 (10.3)
Cronbach's alpha‖			*0.80*		

Abbreviations: AIDS, acquired immunodeficiency syndrome; TB, tuberculosis.
†: Some people (18 for the overall population, 12 for male, and 6 for female) were missing at least one item of the stigma scale.
‖: Cronbach's Alphas were calculated using the complete cases (231 for the overall population, 147 for people ≤15 years old, and 84 for people >15 years old).

Supplementary Table S4. Correlations between adolescent tuberculosis stigma scale and other key psychosocial indicators among adolescents undergoing tuberculosis treatment in Lima, Peru, stratified by gender†

	γ
**Overall (N=231)**	
Adverse childhood experience	0.13*
Depression	0.39***
Suicidal ideation	0.26***
Self-efficacy	0.01
Social support	-0.19**
**Males (N=147)**	
Adverse childhood experience	0.07
Depression	0.41***
Suicidal ideation	0.23**
Self-efficacy	0.07
Social support	-0.31***
**Females (N=84)**	
Adverse childhood experience	0.23*
Depression	0.30**
Suicidal ideation	0.29**
Self-efficacy	-0.10
Social support	0.04

*: p<0.05; **: p<0.01; ***: p<0.001.
†: Participants who were missing stigma score were excluded from the analysis.

Supplementary Table S5. Confirmatory factor analysis (sensitivity analysis) of adolescent tuberculosis stigma scale among all adolescents undergoing tuberculosis treatment in Lima, Peru (N=249)†

Items	Crude model∆	Modified model*
1. Scared to tell my family members that I have TB	0.64	0.61
2. Scared to go to the health center to get my pills for fear that others will see me	0.66	0.65
3. Scared to tell other people I have TB because they may think I have AIDS	0.63	0.65
4. Guilty that I got TB because I didn't eat well	0.42	0.41
5. Guilty because I have TB and therefore am a burden for my family	0.36	0.33
6. Careful about whom I tell I have TB	0.42	0.38
7. Scared that I will lose my friends if I tell them I have TB	0.65	0.67
8. Scared to tell people outside my family that I have TB	0.67	0.66
9. Alone because I have TB	0.54	0.48
10. Hurt because of the way others have reacted when they find out I have TB	0.67	0.66
Model fits (indicators and reference)		
$X^2/df \leq 3.0$	X^2/df=3.7	X^2/df=2.0
P ≥ 0.05	P<0.001	P=0.001
RMSEA ≤ 0.06	RMSEA=0.10	RMSEA=0.06
CFI ≥ 0.90	CFI=0.82	CFI=0.94
TLI ≥ 0.90	TLI=0.76	TLI=0.92
SRMR ≤ 0.08	SRMR=0.07	SRMR=0.05

Abbreviations: AIDS, acquired immunodeficiency syndrome; TB, tuberculosis.
∆: Model without any modifications.
*: Model fit was improved after specifying the correlations between item 10 and item 6; item 1 and item 2; item 11 and item 10; item 9 and item 7.
†: Missing data was handled using full information maximum likelihood estimation.
